# Predicting provenance of forensic soil samples: Linking soil to ecological habitats by metabarcoding and supervised classification

**DOI:** 10.1371/journal.pone.0202844

**Published:** 2019-07-08

**Authors:** Camilla Fløjgaard, Tobias Guldberg Frøslev, Ane Kirstine Brunbjerg, Hans Henrik Bruun, Jesper Moeslund, Anders Johannes Hansen, Rasmus Ejrnæs

**Affiliations:** 1 Section for Biodiversity, Department of Bioscience, Aarhus University, Rønde, Denmark; 2 Natural History Museum of Denmark, Copenhagen, Denmark; 3 Department of Biology, University of Copenhagen, Copenhagen, Denmark; Oklahoma State University, UNITED STATES

## Abstract

Environmental DNA (eDNA) is increasingly applied in ecological studies, including studies with the primary purpose of criminal investigation, in which eDNA from soil can be used to pair samples or reveal sample provenance. We collected soil eDNA samples as part of a large national biodiversity research project across 130 sites in Denmark. We investigated the potential for soil eDNA metabarcoding in predicting provenance in terms of environmental conditions, habitat type and geographic regions. We used linear regression for predicting environmental gradients of light, soil moisture, pH and nutrient status (represented by Ellenberg Indicator Values, EIVs) and Quadratic Discriminant Analysis (QDA) to predict habitat type and geographic region. eDNA data performed relatively well as a predictor of environmental gradients (R^2^ > 0.81). Its ability to discriminate between habitat types was variable, with high accuracy for certain forest types and low accuracy for heathland, which was poorly predicted. Geographic region was also less accurately predicted by eDNA. We demonstrated the application of provenance prediction in forensic science by evaluating and discussing two mock crime scenes. Here, we listed the plant species from annotated sequences, which can further aid in identifying the likely habitat or, in case of rare species, a geographic region. Predictions of environmental gradients and habitat types together give an overall accurate description of a crime scene, but care should be taken when interpreting annotated sequences, e.g. due to erroneous assignments in GenBank. Our approach demonstrates that important habitat properties can be derived from soil eDNA, and exemplifies a range of potential applications of eDNA in forensic ecology.

## Introduction

In ecological studies, bioindication is routinely used to infer environmental conditions and ecosystem properties, and to classify vegetation types [1–3]. The link between species and environmental conditions is also the basis of the application of ecology in forensic science [4]. In a wide range of disciplines–such as palynology, botany and entomology–pollen, plant fragments or insect remains are identified or analyzed by experts to impart ecological information to criminal investigations [5–8]. Similarly, forensic geoscience builds on the geological disciplines of inorganic soil analysis, i.e., soil classification, mineralogy, soil chemistry and physics [9].

Soil is commonly encountered as trace evidence in criminal cases, i.e. mud sticking to footwear, tires and shovels, soil splash marks on vehicles, and traces left on clothes, the floor or in the trunk of vehicles. Those soil samples can be compared to samples from known locations, where an offence is thought to have occurred, thereby establishing a link between a suspect or a victim and a crime scene. In an investigative process, where for example the crime scene is unknown, soil trace evidence can also give valuable information on geographic origin or provenance and help narrow the search for a location. However, inorganic soil properties tend to vary at regional scales, which limits the precision of soil sample provenance [10], but recent digital signatures from x-ray fluorescence have demonstrated a potential for high precision prediction accuracy for local scale soil provenance [11].

Soil contains DNA from the living (and dead) biota below and above ground. Plants are rooted in the soil, whereas fungi and other microorganisms live in the soil, and animals live, dwell or dig through the soil, leaving DNA traces behind. Even organisms living entirely above ground leave DNA in the soil as they defecate, exude secretions or die and decay. Extracting DNA from soil has been in use for decades in microbiology for characterization of bacterial soil communities [12]. Also, microbial profiles from DNA analysis have been used as a fingerprinting tool in forensic investigations [13, 14]. With the development of metabarcoding of environmental DNA from soil (from here onwards: soil eDNA; [15]), high-throughput sequencing of marker genes allow simultaneous detection of multiple species in a complex DNA extract from a single sample. These methods have rapidly found application in ecology [16], in which DNA metabarcoding data are used in ecological studies and conservation biology to identify communities or assess biodiversity [17, 18]. Recently, these methods have also been employed in forensic ecology. Examples include a “biological signature” of annotated taxa in soil DNA used to describe vegetation characteristics [19] and “fingerprints” of sequence composition used to match forensic soil samples [20]. Besides the latter application of matching forensic soil samples, soil eDNA also has great potential to improve predictions of provenance from trace evidence because species communities define habitats, vary across environmental gradients and geographic distance. Therefore, we address whether soil eDNA can be used to predict a sample’s origin along environmental gradients (light, soil moisture, pH and nutrient status), origin in terms of habitat types (e.g., forest, heathland and rotational field), and in terms of geographic origin. We explored the degree to which expert evaluation of plant sequences could enhance these predictions. Lastly, we demonstrate the potential application of provenance prediction in forensic science by evaluating two case studies in detail.

## Methods

### Sample sites

This study is based on field data collected in the project “Biowide” (project website with site locations: http://bios.au.dk/om-instituttet/organisation/biodiversitet/projekter/biowide/), a large nation-wide survey of biodiversity in Denmark, where multitaxon biodiversity and its potential drivers were investigated within the ecospace framework [21]. Biowide includes 130 study sites (40 m × 40 m) evenly distributed across five geographic regions in Denmark. Within each region, sites were placed in three clusters for logistical reasons, but with a minimum distance of 500 m. Site selection was stratified according to primary environmental gradients. Thirty sites were allocated to cultivated habitats and 100 sites to natural habitats. The cultivated subset was stratified according to major land-use types and the natural subset was stratified according to gradients in soil nutrient status, soil moisture and successional stage. Saline and fully aquatic habitats were deliberately excluded, but temporarily inundated depressions as well as wet mires and fens were included. The final set of 24 environmental strata consisted of the following six cultivated habitat types: Three types of fields (rotational, grass leys, set aside) and three types of plantations (beech, oak, spruce). The remaining 18 strata were natural habitats, constituting all factorial combinations of: fertile and infertile; dry, moist and wet; open, tall herb/scrub and forest. These 24 strata were replicated in each of the five geographical regions. We further included a subset of 10 perceived hotspots for biodiversity in Denmark, selected subjectively by public voting among active naturalists in the Danish conservation and management societies, but restricted so that each region held two hotspots. See Brunbjerg *et al*. [22] for a thorough description of site selection and stratification. All analyses were performed on data from the 130 sites, i.e., all provenance predictions were made for the 130 sites using leave-one-out model prediction. To present and discuss the results, we looked through the results from 20 randomly chosen sites and from those we chose two sites (one forested and one open habitat) for which we will present and discuss the provenance predictions in depth, i.e., our two “mock crime scenes”. The study sites were located both on privately and state owned land. The Danish Agency for Nature issued permission for state-owned land and all private landowners were contacted and gave permission to do the field work on their land. All field work and sampling was conducted in accordance with Responsible Research at Aarhus University and Danish law, i.e., no further permissions were necessary for the data sampling. The data presented here does not include sampling of endangered or protected species. Plant species nomenclature follows the database https://allearter.dk/.

Soil samples were collected in Biowide and Ellenberg Indicator Values (EIV, [23]) were calculated based on plant inventories from Biowide. Collection of soil for eDNA metabarcoding was also part of Biowide, but several datasets are published here for the first time, and for already published sequence data improvements were made in the bioinformatics as part of the current study. All statistical analyses and applications of the data are novel for the present study.

### Soil eDNA metabarcoding

Also, as a part of Biowide, soil samples were collected from all 130 sites and subjected to eDNA metabarcoding through DNA extraction, PCR amplification of genetic marker regions (DNA barcoding regions) and massive parallel sequencing on the Illumina MiSeq platform as described in Brunbjerg *et al*. [22]. For this study, we used sequencing data from marker genes amplified with primers targeting eukaryotes (nr18S), fungi (nrITS2), plants (nrITS2) and insects (mt16S). The soil sampling scheme included the mixing of 81 core-like soil samples from each site (9 x 9 samples in the 40 x 40 m site) in an attempt to get a representative bulk sample. Soil samples were collected at c. 0–15 cm depth with a thistle remover gardening tool with a curved open blade (Wolf-Garten, iW-M 2553000) into a barrel (CurTec 15 L Wide neck drum, HDPE). Larger roots and top litter layer was removed. Volume of bulk soil sample was c. 10 L but varied slightly according to soil type. Each bulk sample was mixed and homogenized with a drilling machine (HILTI Cordless Combihammer) mounted with a mixing paddle and 4 g was subsampled for DNA extraction. DNA was extracted with the PowerMax Soil DNA Isolation kit (MOBIO, Carlsbad, CA, USA) after addition of 4 mL of 1M CaCO_3_ suspension. DNA extract was purified with PowerClean DNA Clean-Up Kit (MOBIO, Carlsbad, CA, USA), and normalized to 1 ng/μl after initial fluorometric quantification using the Qubit dsDNA HS Assay Kit. For eukaryotes we amplified part of the nuclear ribosomal (small subunit) region (nr18S) with primers 18S_allshorts with a slight modification of the forward primer (TTTGTCTGGTTAATTCCG). The nuclear ribosomal internal transcribed spacer region ITS2 (nrITS2) was amplified with primers gITS7 and ITS4 targeting fungi, and also with S2F and ITS4 targeting vascular plants. The mitochondrial 16S region (mt16S) was simplified with primers Ins_F and Ins_R [24] intended to target primarily insects. PCR amplifications contained 1X AmpliTaq Gold (Life Technologies), 0.625 μM of each primer, 0.83 mg/ml bovine serum albumin (BSA) and 1,5 μL DNA extract, 1X Gold Buffer, 2.5 mM of MgCl2, 0.08 mM each of dNTPs in 24 μL reaction volume. Primers were designed with 80 unique tags (MID/barcodes) of 8–9 bp at the 5’ end. No primer tag was used more than once in any sequencing library and no combination of forward and reverse primer was reused in the study. Each sample was amplified three times. PCR products were pooled for a total of 6 pools per marker. PCR pools were cleaned with MinElute purification kit (QIAGEN GmbH). The 6 pools were built into separate sequencing libraries using the TruSeq DNA PCR-Free Library Preparation Kit (Illumina). Adapter dimers were removed using Agencourt AMPure XP beads. Sequencing was carried out on a MiSeq (Illumina Inc., San Diego, CA, USA) at the Danish National Sequencing Centre using 250 bp PE runs, two runs for the fungi.

The bioinformatic processing of the sequence data followed the strategy outlined in [22]. Demultiplexing of samples was done with custom scripts that keeps R1 and R2 separate for DADA2 processing [25], and is based on Cutadapt and Sickle for the fungi and plant data (see [26]). We used DADA2 (v 1.8) [25] to identify exact amplicon sequence variants (ESVs) and for removal of chimeras (bismeras). Sequences were filtered and matched between R1 and R2 reads with DADA2. The DADA2 table was used without further modifications for the nr18S (eukaryote) as the employed primers were expected to only amplify eukaryotes. An initial screening of the mt16S data showed that the employed primers amplify many groups other than insects, and that many ESVs could not be assigned to any lineages, and thus, that a filtering to insects alone would mean discarding most of the data, so we decided to include the full DADA2 table without further modifications in the following analyses. For the plants we wanted to be able to use species level information in the analyses and thus performed a more elaborate operational taxonomic unit (OTU) definition and taxonomic assignment and filtering, comparable to that performed for the fungal dataset [26]. Sequences were extracted with abundance information sample wise with a custom script, the ITS2 region was extracted ITSx [27], and clustering into OTUs was done with VSEARCH (v 2.3.2) [28] at 98.5% similarity for fungi and 97% for plants. Post-clustering curation using LULU [29] eliminated remaining redundant sequences for the plant and fungi. The LULU match list was made with blastn and (using options: -qcov_hsp_perc 80 -perc_identity 84), and LULU (with options minimum_match = 84, minimum_relative_cooccurence = 1). OTUs flagged as errors by LULU were discarded unless they had a reference database match of ≥ 98.5% and had a non-redundant taxonomic annotation. OTUs were taxonomically annotated by blasting against GenBank (nt) with blastn (option qcov_hsp_perc 70), and perc_identity 60 for plants, and for fungi by matching against v8.0 UNITE general FASTA release with vsearch (vsearch—usearch_global—dbmask none—qmask none—query_cov .98—maxaccepts 0—maxrejects 0—top_hits_only—maxhits 1—id 0.5—iddef 2), and the annotation was adjusted to the match percentage with reference database using 98%, 90%, 85%, 80%, 75% and 70% sequence identity for assigning OTU to species, genus, family, order, class or phylum, respectively. Taxonomic affiliation of the plants was assessed with a custom script assessing the most widely applied name among the top hits for plants, and for fungi by using the best match in UNITE. Data from pcr replicates (3) was merged. OTUs that could not be assigned to the target group for the fungal dataset (kingdom Fungi) and the plant dataset (phylum Streptophyta, but excluding classes Chlorophyta, Sphagnopsida, Jungermanniopsida, Bryopsida and Polytrichopsida), were removed from these datasets. Documentation of the bioinformatic processing and links to the sequencing data can be found on GitHub (github.com/tobiasgf/provenancing). The fungal sequence data and bioinformatic processing of this was originally published in Frøslev et al. [26], and the plant sequence data was published in Frøslev et al. [29], where it was analyzed in a slightly different way. Due to the unfiltered mt16S dataset we will from here on after refer to the OTU datasets as Plants, Fungi, Eukaryotes and mt16S-’insects’.

To obtain meaningful predictors from soil eDNA for this study, we did NMS-ordinations for each of the four OTU community datasets (Fungi, mt16S-‘insects’, Eukaryotes and Plants) using abundance data (number of sequences per OTU) with square root transformation followed by Wisconsin double standardization. The output of each ordination is four axes essentially representing the variation in OTU composition between all sites, i.e., the axes of the ordination of fungi OUT community data results in 4 axes representing major gradients in fungi community composition across all sites. Hence we used these 4 sets of 4 axes as explanatory variables in our statistical analyses.

### Predicting environmental gradients

As response variables for environmental gradients, we used community mean Ellenberg Indicator Values (EIV, [23]). The validity of plant-based bioindication has been confirmed by direct measurement of the environmental conditions and by plant growth experiments (e.g., [30, 31]) and is an approach commonly used in vegetation studies to assess local environmental conditions (e.g., [32]). EIVs for light, soil moisture, pH and nutrient status (EIV L, M, R and N) were calculated in Biowide. Each plant species is assigned an EIV [33] and based on plant species lists from a site, the community or site EIV is calculated as an average of all the indicator values assigned to each plant species in the community.

First, we used linear models to evaluate the relationship between OTU composition (the first four NMS axes) and environmental gradients. Model selection was based on AIC (Akaike Information Criterion) using the function stepAIC and backwards selection in R package MASS [34]. Individual models were constructed for each set of NMS axes for the different taxonomic groups and normality and heterogeneity were assessed by visual inspection of residual plots. The final model for EIV light showed some non-linear patterns and we checked if a GAM ([35], default settings and k = 3) improved AIC and model fit, which it did and a GAM was chosen instead of the linear model for light. Second, the best set of predictors (NMS axes) was selected to predict environmental gradients and standard error using leave-one-out cross validation. All analyses were performed in R-3.4.2 [36].

**Fig 1:**
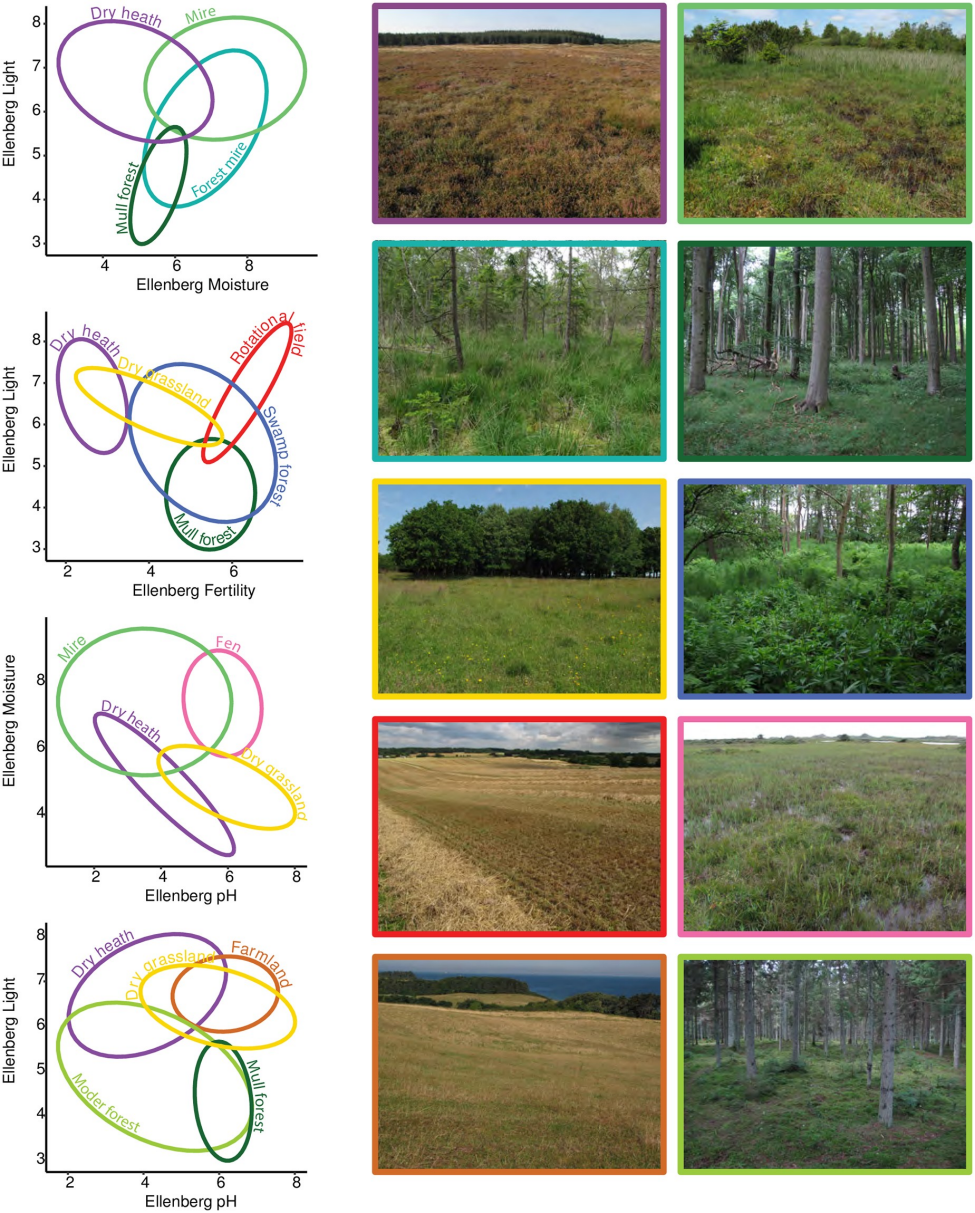
Interpreting Ellenberg Indicator Values. Ellipses showing the multivariate normal distribution of vegetation types plotted along Ellenberg indicator values for light, soil moisture, pH and nutrient status. Agricultural includes rotational fields, old fields and lays. The colors of the ellipses correspond to characteristic sites depicted on the right.

While most trained botanists and ecologists may be familiar with EIV, to most people they are hard to interpret in terms of meaningful vegetation types of environmental conditions. We have therefore aided interpretation by graphing the location of common vegetation types along the four environmental gradients used in this study (Fig 1).

### Predicting binary habitat types

Another aim was to describe habitats typical of Denmark from the OTU composition in soil samples. For this study, we selected habitat types that are familiar to most people, easy to identify by non-ecologists and possible to recognize from a distance or from publicly available maps and orthophotos with some training (Table 1). The habitat types were defined and recorded as binary variables for each site after plant inventories, but before statistical analysis, i.e., it is an a priori classification.

**Table 1:** Habitat types and geographic origins and their description.

**Habitat type**	Explanation
High forest	High forest, characterized by tall trunks and with an average canopy height ≥ 9 m (n = 23)
Forest	Forests including woodlands and tall shrubs with and average canopy height ≥ 2 m (n = 53)
Agriculture	Rotational field, grass ley and fallow field (n = 14)
Dwarf shrub	Dominance of dwarf shrubs (*Vaccinium*, *Calluna*, *Erica*, *Empetrum*, etc.), but both forest and open vegetation types (n = 28)
Beech	Forest with *Fagus sylvatica* dominance (n = 17)
Oak	Forest with deciduous *Quercus* (mainly Q. robur) dominance (n = 11)
Willow	Tall shrubland or low forest on moist to wet soils dominated by *Salix* spp. (n = 10)
Coniferous	Forest and tall shrubland dominated by coniferous trees and shrubs (*Abies*, *Picea* or *Juniperus*) (n = 9)
Heathland	Northern Atlantic wet heath with *Erica tetralix* OR European dry heath (also known as the habitat types 4010 and 4030 on the EU Habitats Directive, [37]) (n = 6)
Alder	Swamp forest with *Alnus* dominance (n = 15)
Reed swamp	Wetlands dominated by *Phragmites australis* (n = 18)
**Geographic origin**	Explanation
Atlantic	Located in the Atlantic biogeographic region of Denmark (as opposed to the Continental region) (n = 35)
Jutland	Located in Jutland, the mainland of Denmark (as opposed to the Islands, i.e., Funen, Zealand, Lolland and Møn) (n = 78)

The habitat types are binary, i.e., the characteristic is either present or absent at a site.

The habitat types were modelled using Quadratic Discriminant Analysis (QDA) using the function qda in package MASS [34, 38] and leave-one-out cross validation was used for model selection and estimation of prediction error. The explanatory variables were the previously mentioned set of NMS axes of the four different OTU-groups (eukaryotes, fungi, plants and mt16S-‘insects’). Model selection was performed by adding variables that improved the percentage correctly predicted in the cross validation. We used the class proportions of class or habitat membership for the training set as prior probabilities. As the distribution of the binary characteristics is often skewed with few sites with class membership = 1, the percentage correctly predicted tends to be high solely because of a high percentage true negatives. Therefore, we also evaluated model performance on the percentage of true positives.

We also investigated the possibility of predicting geographic origin defined as a binary response variable, i.e., mainland/islands and atlantic/continental biogeographic regions of Denmark, using the above-mentioned predictors.

The best model was then selected based on the highest percentage correctly predicted and a percentage of true positives > 50%. When the percentage correctly predicted was very similar, we chose the model with highest percentage true positives.

For the habitat types that were characterized by one or a few species, we tested if by adding the frequency of sequences annotated to those plant taxa as an additional explanatory variable improved the model fit. We only used OTUs with a taxonomically unambiguous reference database (GenBank) match ≥ 98.5%. Based on our knowledge of the species pool in Denmark, the frequency of sequences for the following taxa were added as additional explanatory variables to the best models: *Fagus* for beech forest, *Quercus* for oak forest, Pinaceae for coniferous forest, Ericaceae for heathland, *Alnus* for alder swamp, Salix for willow shrubs and *Phragmites australis* for reed swamp.

### Looking for rare or geographically indicative species

Most plant species are somewhat rare, i.e., either confined to a rare habitat type or geographically confined and–in the latter case–considered potentially useful for assessing the geographic provenance of a soil sample. OTUs of rare plant species may occur at single sites in the dataset or with very low relative sequence abundance and are therefore not likely to drive geographic patterns in the ordination of the whole dataset. Instead, we targeted these species in a separate exercise. For this approach, we only used OTUs with a taxonomically unambiguous reference database (GenBank) match ≥ 98.5%. We used the nomenclature in Genbank (NCBI taxonomy). Among the unambiguously annotated plant species we looked for rare species, i.e., occurring in less than 224 Atlas Flora Danica grid cells, corresponding to c. 10% of the cells in the Atlas Flora Danica survey [39], a national mapping of plant species in Denmark in 5 × 5 km grid cells. In addition, we identified plants, which are regionally or locally confined in their distribution (S1 Table). Cultivated plants, such as common crops, may also help predict provenance of soil samples and can be compared to the national mapping of fields and crops. For example, we looked for sequences annotated to *Triticum* species, *Brassica napus*, *Beta vulgaris*, *Secale cereale*, and *Zea mays*.

All data and analysis can be found on GitHub (github.com/tobiasgf/provenancing).

## Results

Axes from an NMS ordination of OTU communities were good predictors of environmental gradients of light, soil moisture, pH and nutrient status as represented by EIVs (0.81≤R^2^≤0.89 for the best models, Table 2). The predictive ability of NMS axes based on the different taxonomic target groups were examined separately and fungal OTUs were the best predictors of light, soil moisture and pH, whereas plant OTUs best predicted soil nutrient status (Table 2).

**Table 2:** Predicting environmental gradients of light, soil moisture, pH and nutrient status from variation in soil eDNA.

EIV	Fungi	mt16S-‘Insects’	Eukaryotes	Plants
Light	0.76	0.59	0.55	0.65
Light (GAM)	**0.81**	-	-	-
Moisture	**0.84**	0.80	0.84	0.78
pH	**0.89**	0.75	0.79	0.72
Nutrient	0.82	0.64	0.71	**0.83**
N	130	130	130	130

Model R^2^ values of linear models with EIVs of light, soil moisture, pH and nutrient status as response variable and NMS ordination axes of soil OTU community composition of fungi, insects, eukaryotes and plants as explanatory variables. Text in bold indicates the best model for each environmental gradient used for prediction.

NMS axes also showed good discriminatory power as predictors of habitat types in QDA (Table 3). Based on leave-one-out cross validation, the best models for each habitat type performed well, with >89% correctly predicted sites. The percentages of true positives for the best models were > 53%. Models did not perform well for Heathland and the Atlantic region and these were therefore not used for predicting provenance. We found a total of 304 unambiguously annotated plant species of which 37 species are rare, i.e., occurring in less than 224 Atlas Flora Danica grid cells, corresponding to c. 10% of the cells in the Atlas Flora Danica survey [39]. In addition, we identified 41 plants with distinct regional or local confined distributions (S1 Table and S1 Fig). In 98 out of the 130 samples we found sequences of at least one of the 78 regionally or locally distributed species. Four samples included sequences annotated to common crops of *Triticum*, *Beta vulgaris* and *Secale*.

**Table 3:** Predicting habitat types from variation in soil eDNA.

Habitat type	Fungi	mt16S ‘insect’	Eukaryotes	Plants	With sequences
HighForest	**0.90 (0.83)**	0.90 (0.78)	0.82 (0)	0.89 (0.74)	-
Forest	**0.95 (0.96)**	0.85 (0.81)	0.94 (0.92)	0.92 (0.83)	-
Agriculture	0.92 (0.64)	0.95 (0.5)	0.92 (0.71)	**0.98 (0.93)**	**-**
Coniferous	0.93 (0.00)	0.93 (0.00)	0.93 (0.00)	0.93 (0.00)	0.96 (0.44)
Beech	**0.96 (0.82)**	0.92 (0.71)	0.87 (0)	0.95 (0.88)	0.95 (0.76)
Oak	**0.92 (0.55)**	0.92 (0.00)	0.92 (0.00)	0.92 (0.00)	0.95 (0.45)
Willow	**0.96 (0.70)**	0.92 (0.00)	0.92 (0.00)	0.93 (0.30)	0.95 (0.60)
Heathland	0.95 (0.00)	0.95 (0.00)	0.95 (0.00)	0.95 (0.00)	0.92 (0.50)
Dwarf shrubs	**0.91 (0.82)**	0.78 (0.00)	0.89 (0.71)	0.87 (0.75)	-
Alder	**0.90 (0.53)**	0.88 (0.00)	0.9 (0.47)	0.9 (0.20)	0.93 (0.42)
Reed swamp	0.88 (0.50)	**0.89 (0.56)**	0.89 (0.50)	0.9 (0.39)	0.88 (0.18)
Atlantic	0.73 (0.00)	0.77 (0.26)	0.73 (0.00)	0.73 (0.00)	-
Jutland	0.65 (0.90)	**0.75 (0.79)**	0.66 (0.91)	0.62 (0.86)	-

The percentage of correctly predicted binary habitat types from quadratic discriminant analysis (QDA) with NMS ordination axes of soil OTU community composition of fungi, insects, eukaryotes and plants as explanatory variables and after variable selection. Percent true positives are reported in parenthesis. Column “with sequences” shows results for habitats characterized by a certain taxa, in which case we tested whether adding the frequency of sequences of that taxa as an additional explanatory variable would improve model fit of the best model. The “-”sign shows that sequences were not added the best model as that habitat type is not characterized by one specific taxonomic group. Text in bold indicates the best model selected. When model performances are equal, we opt for the simpler model.

**Fig 2:**
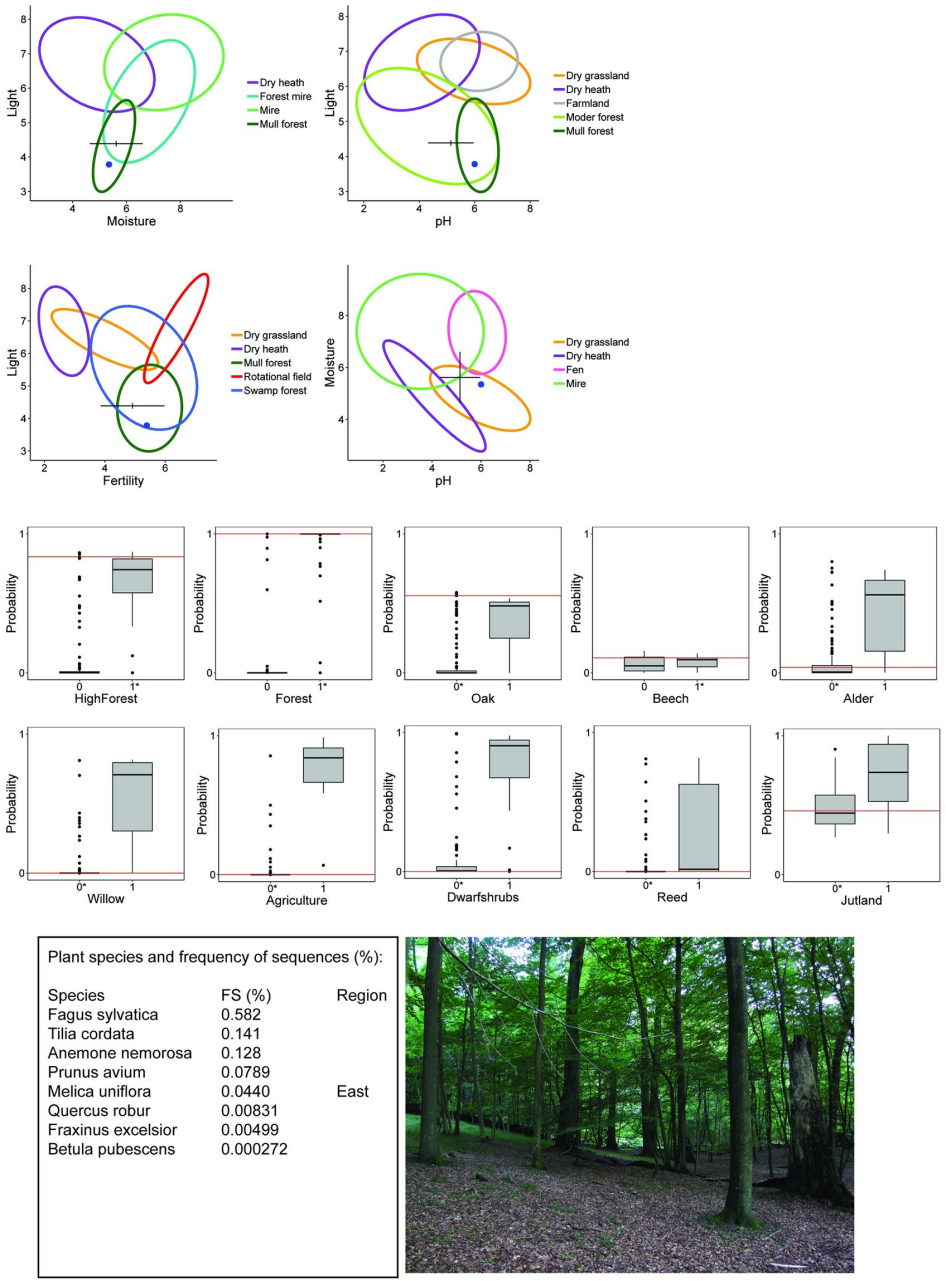
Mock crime scene 1 and the provenance derived from soil eDNA. Predicted location in environmental space (Ellenberg Indicator Values) based on the best linear models and GAM for light (top left). The cross marks the predicted EIV value and the length of the arms show the 95% confidence intervals. Blue dots show the actual EIVs at the sample site. The predicted probability of binary habitat types based on Quadratic Discriminant Analysis are shown as red lines on a box plot of the distribution of predicted values for each characteristic (middle part). A priori classification membership is indicated by asterisk of the 0/1 variable. The probabilities for heathland, coniferous and Atlantic are not shown. A list of plant species sequences, their frequency of sequences (FS) in the sample, and if applicable, the geographic region of their distribution in Denmark is listed bottom left. For evaluation, we provide a picture of the sample location (bottom right).

**Fig 3:**
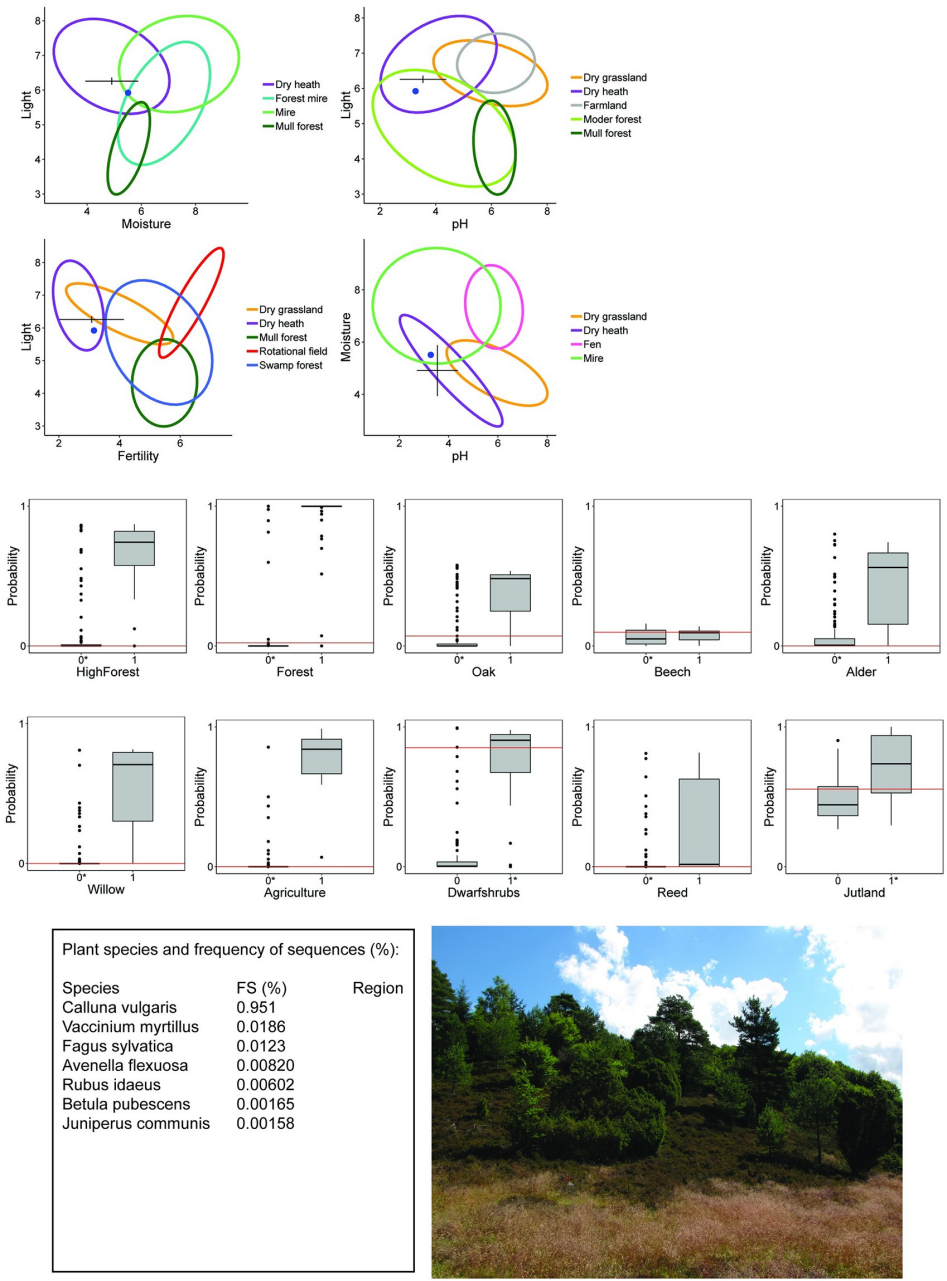
Mock crime scene 2 and the provenance derived from soil eDNA. Predicted location in environmental space (Ellenberg Indicator Values) based on the best linear models and GAM for light (top left). The cross marks the predicted EIV value and the length of the arms show the 95% confidence intervals. Blue dots show the actual EIVs at the sample site. The predicted probability of binary habitat types based on Quadratic Discriminant Analysis are shown as red lines on a box plot of the distribution of predicted values for each characteristic (middle part). A priori classification membership is indicated by asterisk of the 0/1 variable. The probabilities for heathland, coniferous and Atlantic are not shown. A list of plant species sequences, their frequency of sequences (FS) in the sample, and if applicable, the geographic region of their distribution in Denmark is listed bottom left. For evaluation, we provide a picture of the sample location (bottom right).

We present two mock crime scenes as examples of how these predictions can be collated and presented to investigators in a meaningful way (Figs 2 and 3). For mock crime scene 1, the predicted EIVs placed the site at an intermediate position for soil pH and moisture, slightly fertile soil and low light conditions. Considering all four environmental predictions and with the aid of the ellipses in Fig 1, this indicated mull or moder forest site. The prediction of binary habitat are presented as bar plots of all the predicted probabilities of the habitat type, i.e., clearly vertically separated bars indicates that the model performs well in distinguishing between habitat type membership. The red line shows the predicted probability of the mock crime scene, i.e. the higher the red line, the higher the probability of belonging to that habitat type. In this case, there were very low probabilities for open vegetation types (e.g., agriculture and dwarf shrubs) and wet vegetation (e.g., reed swamp, willow and alder dominated habitat types) and high probabilities for the habitat types forest, high forest and oak, and low probability that the location is in Jutland. The list of plant species and their frequency of sequences in the sample showed that *Fagus sylvatica*, *Tilia cordata* and *Anemone nemorosa* were very frequent and accompanied by other common forest plant species, such as *Melica uniflora* and *Quercus robur* (Fig 2). The geographic distribution of *M*. *uniflora* in Denmark is limited to the eastern parts (i.e., the continental region, S1 Table) and corresponds to the low prediction for Jutland, which is in the west. The mock crime scene in this case was an old growth beech forest on moder soil in Zealand, Eastern Denmark.

Predicted EIVs for mock crime scene 2 indicated intermediate light conditions, low nutrient status, low soil pH and relatively low soil moisture, which was within the ellipse of dry heathlands (Fig 3). The binary predictions showed low probability for forest and high forest. Low probabilities for other forest characteristics indicated that it was unlikely to be a characteristic beech, oak, or coniferous forest. There were high probabilities for dwarf shrubs, which corresponded to the annotated species found in the soil sample and relatively high probability for the location being in Jutland. The site OTU community had a very high relative abundance of *Calluna vulgaris* sequences and less of *Vaccinium myrtillus*, *F*. *sylvatica Avenella flexuosa*, *and Rubus idaeus*. The mock crime scene 2 is a dry heathland with scattered trees (*F*. *sylvatica*, *Q*. *robur* and *Pinus sylvestris)* and *Juniperus communis* shrubs. The site is located in Jutland in Western Denmark.

## Discussion

We investigated the potential for constructing predictive models of environmental properties, habitat types and geographic origin based on soil eDNA. We found that variation in soil eDNA predicts environmental conditions and most habitat types well and geographic provenance less so. The latter result corresponds to ecological studies showing that, within habitat types, geographic variation in species composition is limited within Denmark (e.g., [40]). Previous attempts to use eDNA to predict geographic provenance are few and the scale of prediction is important to explore. DNA sequences of fungi in dust have been shown to predict geographic origin with a few hundred km precision at a continental scale [41]. For comparison, Denmark extends c. 300 km from east to west. Recently, spectral analysis–another discipline of forensic geoscience–was shown to successfully predict geographic origin at local scales in a cultural landscape [11]. It is possible that eDNA also will perform better at predicting local scale provenance within a specific urban landscape, which may be characterized by novel communities and introduced species [42].

Variation in eDNA predicted environmental gradients, i.e., EIVs, in linear models with R^2^ >0.81. NMS-axes based on different genetic markers produced good models, but fungi produced the best models for light, soil moisture and pH and a good model for nutrient status indicating that it may be more efficient to focus on fungi primers for this type of analysis. Here, we used EIVs for light, soil moisture, pH and nutrient status [23]–the validity of which has been confirmed by direct measurement of the environmental conditions and by plant growth experiments (e.g., [30, 31, 43]). EIVs are, however, available only for the Central European and British flora, but for application in other parts of the world, EIVs may be replaced by species scores from ordination of large and representative vegetation datasets, which typically reflect major environmental gradients [44].

While habitat types are often described and delimited by distinct characteristics and plant communities [37], they are, in reality not discrete entities, but instead fuzzy and overlapping in species composition along continuous environmental and successional gradients. Often plant communities occur in patchy mosaics. Performance of our classification models tended to be best for habitats that are relatively well defined and delimited along such gradients, e.g., forest. At the other end of the spectrum, models performed relatively poorly for heathland, which we defined broadly to include both wet and dry heathland with a variable degree of tree and shrub cover. Similar communities can be found in mires, grasslands and plantations (see also the overlap in the ellipses in Fig 1), making accurate prediction of heathland difficult.

Two mock crime scenes showed that model predictions for environmental conditions corresponded well with the actual EIVs at the site. EIVs are commonly used in vegetation studies to assess local environmental conditions (e.g., [32]). The validity of such plant-based bioindication of environmental conditions has been confirmed by direct measurement of the environmental conditions and by plant growth experiments (e.g., [30, 31]).

Predictions of habitat types did not always correspond to the *a priori* classifications at a first glance, but this could reflect the continuous nature of biotic gradients rather than model failure. For example, mock crime scene 2 is a *Juniperus communis* formation on heathland (5130 on the Habitats Directive) and therefore not classified as a heathland, but as the coniferous habitat type by the definitions used here. However, from the photograph, it is evident that the site bears high similarity to heathland and less similarity to coniferous forests and plantations.

As the models use ordination axes from OTU communities, they are probably less likely to be influenced by mistakes and biases in sequencing and free from mistakes that may arise from annotating sequences to species. While botanists and ecologists can interpret a lot of habitat characteristics and environmental conditions from a plant species list, particular care must be taken when that species list arises from DNA sequence matches with sequence data from public databases, as these may be misidentified or in other ways erroneously annotated (e.g., [45]), or the variation in the marker gene regions may be insufficient to discriminate closely related species. Thus, using plant species for further information requires not only expert knowledge of plant ecology, but also of sequencing, bioinformatics and data quality of reference data. In forensic cases and in the absence of experts, however, care must be taken when interpreting automatically annotated OTUs as there can be biases or mistakes in the database entries, insufficient genetic variation in the marker gene to reliably distinguish specific groups of species, or OTUs with low relative abundance of sequences as these could stem from contamination. For real case forensic work, sequence annotations (and not merely the OTU composition) to infer ecological provenance, should ideally rely on a dedicated reference database of sequences from relevant regional plants, and only unambiguous species matches should be used in the ecological inference to ensure validity, accuracy and reproducibility. Applying universal standards for data collection and sequence curation and interpretation are essential to ensure that the dedicated database adheres to international rules and the latest technologies.

For further work and application of provenancing of soil samples, a national database of soil characteristics including soil eDNA composition is essential. For forensic investigations an obvious disadvantage of the present study is the lack of urban areas, i.e., parks, rubbish dumps, roadsides etc. among the 130 reference samples. However, the dataset is a good starting point as the sites were stratified according to the spectrum of environmental variation in in Denmark. Our results combine expert interpretation of both statistical methods and botanical and ecological data. However, for conclusions to be used in criminal court, likelihood ratios are often preferred, e.g., as already widely used for human DNA matching, and this is of course a point to pay attention to as we develop provenancing.

## Conclusions

Sampling and analyzing soil eDNA by metabarcoding allows interpretation of major environmental gradients and habitat classes relevant for both basic and applied ecology, such as forensic ecology. It demonstrates a new application of eDNA and the basic ecological information that can be extracted from eDNA and variation in OTU assemblages. While we demonstrate the potential application of this technique for predicting and interpreting information relevant to forensic investigations, it is also important to note a number of issues that will improve the application of this predictive tool in forensic investigations. As already mentioned, the present dataset (Biowide) was originally designed to explore biodiversity in natural habitats across Denmark, and as such, urban areas are not represented and agricultural fields and other cultural areas are underrepresented. We know little about the seasonal variation in eDNA (but see e.g., [46]) and variation in eDNA with soil depth (see e.g., [47]). Moreover, forensic soil samples can be minute and contaminated, dried, old and degraded, and we need to explore the model sensitivity and provenancing accuracy of such samples (see also [10]).

Here we demonstrate a new application of eDNA and show that the generated results can be used for sample provenancing relevant to forensic investigations. Hence, the eDNA approach will be a useful investigative tool in crime scene cases without the need for the strict and validated procedures necessary for comparisons that have to be used as evidence in court. Given the generic structure of the eDNA approach we expect it to have global relevance by being applicable in a number of countries outside Denmark.

## Supporting information

S1 Table List of plant species with geographically limited distribution. List of annotated sequences from GenBank, which match rare plants in Atlas Flora Danica [39] and plants with geographically limited distribution (from expert opinion). Abbreviation in Region indicates the regional distribution, i.e., AFDrare, found in less than 10% of Atlas grid cells, N, found in northern Denmark, S, found in Southern Denmark, E, found in eastern Denmark, and W, found in western Denmark.
(DOCX)

S1 Fig Geographic pattern of sample sites containing eastern, western and south-eastern plants. Geographic pattern of sample sites with sequences annotated to plants with geographically limited distributions to western, eastern or south-southeastern Denmark. Green is E, Blue is S/SE and red is W. Size of the circles indicates the number of geographically limited plants.
(TIF)
