# Supplementary material for: Predicting provenance of forensic soil samples: Linking soil to ecological habitats by metabarcoding and supervised classification
Source: PLoS One. 2019 Jul 8;14(7):e0202844. doi: 10.1371/journal.pone.0202844 (PMC6613677; doi:10.1371/journal.pone.0202844)
Supplement: S1 Table — List of annotated sequences from GenBank, which match rare plants in Atlas Flora Danica [39] and plants with geographically limited distribution (from expert opinion). Abbreviation in Region indicates the regional distribution, i.e., AFDrare, found in less than 10% of Atlas grid cells, N, found in northern Denmark, S, found in Southern Denmark, E, found in eastern Denmark, and W, found in western Denmark. (DOCX) [file pone.0202844.s001.docx]

| Species | Distribution | Species | Distribution | Species | Distribution |
| --- | --- | --- | --- | --- | --- |
| *Agrimonia eupatoria* | E | *Cucumis sativus* | AFDrare | *Paris quadrifolia* | E |
| *Alchemilla glaucescens* | AFDrare | *Drosera intermedia* | AFDrare | *Pedicularis palustris* | AFDrare |
| *Allium cepa* | AFDrare | *Eleocharis quinqueflora* | AFDrare | *Pedicularis sylvatica* | AFDrare |
| *Allium scorodoprasum* | E | *Erica tetralix* | W | *Petrorhagia prolifera* | AFDrare |
| *Allium vineale* | coastal | *Euonymus europaeus* | SE | *Platanthera chlorantha* | AFDrare |
| *Andromeda polifolia* | AFDrare | *Filipendula vulgaris* | E | *Poa compressa* | E |
| *Anemone hepatica* | E | *Fragaria viridis* | SE/AFDrare | *Potamogeton polygonifolius* | W/AFDrare |
| *Anemone ranunculoides* | E | *Gagea spathacea* | SE | *Potentilla anserina* | AFDrare |
| *Anthoxanthum aristatum* | AFDrare | *Galium odoratum* | E | *Primula veris* | E |
| *Arnica montana* | W/AFDrare | *Geranium sanguineum* | SE-NW | *Quercus petraea* | AFDrare |
| *Astragalus glycyphyllos* | E | *Hedera helix* | E | *Rhamnus cathartica* | SE |
| *Brachypodium sylvaticum* | E | *Hordelymus europaeus* | SE | *Sagina nodosa* | AFDrare |
| *Campanula persicifolia* | E/AFDrare | *Hypochaeris glabra* | W/AFDrare | *Sanicula europaea* | E |
| *Cardamine bulbifera* | SE/AFDrare | *Inula conyza* | lokal | *Scheuchzeria palustris* | AFDrare |
| *Carex appropinquata* | E/AFDrare | *Juncus alpinoarticulatus* | AFDrare | *Scorzonera humilis* | N |
| *Carex arenaria* | W (and coastal) | *Juncus gerardii* | coastal | *Selinum carvifolia* | E/AFDrare |
| *Carex diandra* | AFDrare | *Juncus squarrosus* | W | *Silene nutans* | E/AFDrare |
| *Carex dioica* | AFDrare | *Lathyrus linifolius* | W | *Stellaria holostea* | E |
| *Carex elongata* | E/AFDrare | *Leontodon hispidus* | E/AFDrare | *Stellaria nemorum* | E |
| *Carex limosa* | AFDrare | *Littorella uniflora* | W/AFDrare | *Tilia cordata* | SE/AFDrare |
| *Carex paniculata* | E | *Melica uniflora* | E | *Trientalis europaea* | W |
| *Centaurea scabiosa* | NE | *Mercurialis perennis* | E | *Trifolium striatum* | E |
| *Chrysosplenium alternifolium* | E | *Nasturtium officinale* | AFDrare | *Vaccinium vitis-idaea* | W |
| *Chrysosplenium oppositifolium* | SW/AFDrare | *Ononis repens* | AFDrare | *Viburnum opulus* | E |
| *Clinopodium acinos* | E | *Origanum vulgare* | SE | *Vicia sylvatica* | S/AFDrare |
| *Corynephorus canescens* | W | *Ornithopus perpusillus* | W | *Viola hirta* | E/AFDrare |
