# Supplementary figures and images for: Predicting provenance of forensic soil samples: Linking soil to ecological habitats by metabarcoding and supervised classification
Source: PLoS One. 2019 Jul 8;14(7):e0202844. doi: 10.1371/journal.pone.0202844 (PMC6613677; doi:10.1371/journal.pone.0202844)

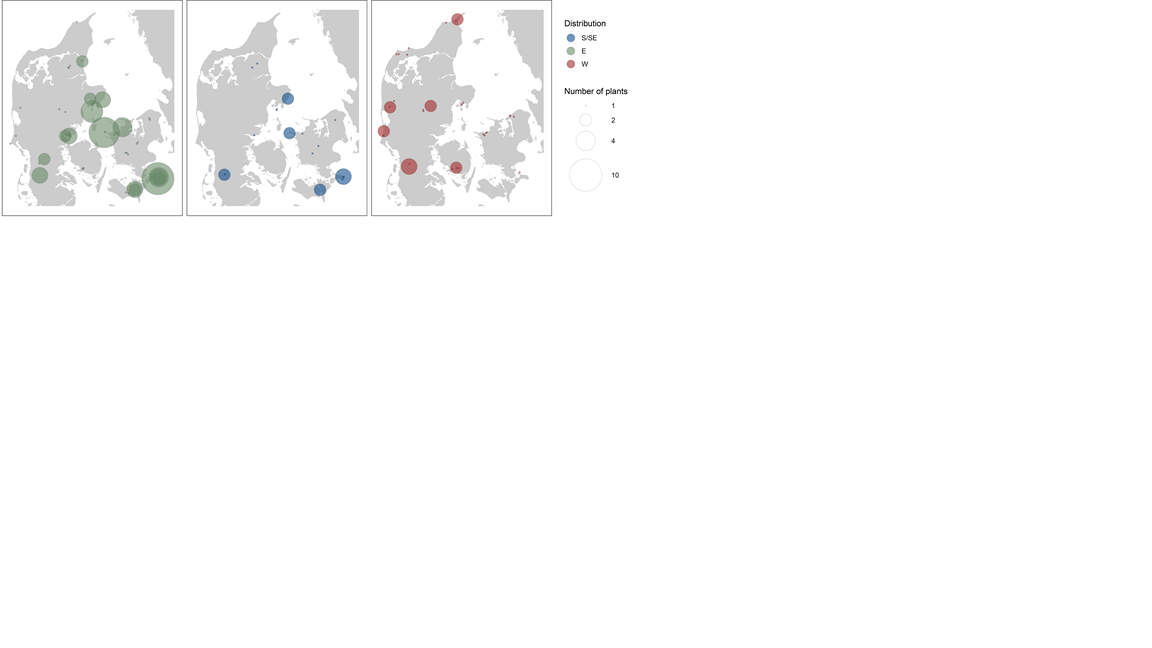

Supplement: S1 Fig — Geographic pattern of sample sites with sequences annotated to plants with geographically limited distributions to western, eastern or south-southeastern Denmark. Green is E, Blue is S/SE and red is W. Size of the circles indicates the number of geographically limited plants. (TIF) [file pone.0202844.s002.tif]
